# Form and function, both matter

**DOI:** 10.1007/s12471-015-0686-5

**Published:** 2015-04-29

**Authors:** W. Mali, P.A. Doevendans

**Affiliations:** 1 Department of Radiology, University Medical Center Utrecht, Heidelberglaan 100, 3584CX Utrecht, The Netherlands; 2 Department of Cardiology, University Medical Center Utrecht, Heidelberglaan 100, 3584CX Utrecht, The Netherlands

In the last decades, the focus of vascular research has been on acute vascular events. The acute coronary syndrome with its dramatic acute clinical manifestations was in the centre of this whirlwind. The focus was on atherosclerotic plaque located in the intima, stenosis, rupture, acute occlusion and the very successful therapeutic interventions. This has eclipsed the other manifestations of vascular disease: stiffening of the vessel wall and arteriosclerosis. This stiffening leads to diminished windkessel function of the elastic vessels, water hammer damage to sensitive tissues and dysfunction of sensors located in the vessel wall. The effects of this stiffening are in general not acute but manifest themselves in the long and probably very long term.

Many slowly evolving vascular manifestations of disease such as dementia, lacunar cerebral infarctions, chronic limb ischaemia and chronic heart failure could be caused by these mechanisms. The stiffening of the vessel wall is due to a mixture of intima proliferation, fibrosing and calcification, which is mainly located in the media layer of the vessel wall.

Although these two processes of atherosclerosis and arteriosclerosis seem to be separate, they can probably occur sequentially or simultaneously and it is feasible that these processes interact with each other.

The arteriosclerotic process has been mainly investigated with the pulse wave velocity (PWV) technique in hypertensive populations [1]. This ultrasound technique investigates aortic stiffness (carotid-aortic PWV), central pulse pressure and augmentation index, and carotid diameter and wall thickness. Yet, this method only provides us with information on a few selected large vessels. So mainly the aorta, carotid and femoral arteries have been investigated. Due to these limitations, technological improvement is needed to assess the complete vascular pathway up to the level of the end organ where the damage occurs.

In this issue, Kroner et al. [2] use such a versatile magnetic resonance technique. By choosing two different planes of measurements that can be placed everywhere in the body, also peripherally located vascular courses can be measured. Furthermore, this technique can show us end-organ damage and, lastly, this technique can detect vessel wall thickness (VWT) reliably. They report a correlation between PWV and VWT and this thickness is related to end-organ damage in the brain in the form of white matter lesions. The relation between PWV and end-organ damage is not mentioned. Was it not significant? Yet PWV should be the explanation for the relation between VWT and end-organ damage unless VWT is an epiphenomenon. The authors wisely state that the numbers are very limited and the significant difference is reported comparing 6 and 9 patients. Even more striking is the identical score in the 6 patients with a low VWT. Therefore, it is prudent to interpret the results with great care. What is also surprising in the study is the fact that age is not identified as a key factor. It is likely that the study is too small for any firm conclusions.

Kroner et al. looked at the stiffness of the carotid artery from the level of the origin of the common carotid artery up to the level of the petrous channel and found that the thickness of the artery in this pathway nicely correlates with white matter lesions. Yet, there is excellent literature showing that the really important part of the internal carotid artery from the point of view of pulse wave modulation is the carotid syphon, located just beyond the petrous channel running in the cavernous sinus [3]. Stiffening of this syphon by calcification, which occurs in up to 80 % of patients above 65 years [4], has been linked to lacunar infarctions [5], generally considered a manifestation of small vessel disease. It is very well possible that by extending the measurement beyond the syphon, PWV would have correlated better with white matter lesions.

And lastly, why is this information important when the general belief is that we cannot change the process of stiffening? It has indeed long been thought that stiffening of the vessel wall with fibrosis and calcification is an unavoidable inert end stage of ageing and certain arterial diseases. However, it is increasingly becoming clear that the calcification component of stiffening is an active metabolic process very much resembling bone formation. The process of bone formation can be halted or even reversed and experimental medication to reverse fibrosis in the form of angiotensin type 2 receptor agonists is becoming available too. With this therapeutic prospect and to protect both grey and white matter, more knowledge about arteriosclerosis is needed. That knowledge should encompass morphology, histology and function of the vessel wall.

## Funding

None.

## Conflict of interest

None declared.
